# Therapeutic Efficacy and Safety of Percutaneous Curved Vertebroplasty in Osteoporotic Vertebral Compression Fractures: A Systematic Review and Meta‐Analysis

**DOI:** 10.1111/os.13800

**Published:** 2023-07-27

**Authors:** Yan Sun, Yong Zhang, Haoning Ma, Mingsheng Tan, Zhihai Zhang

**Affiliations:** ^1^ Department of Orthopaedics Guang'an Men Hospital, China Academy of Chinese Medical Sciences Beijing China; ^2^ Department of Orthopaedics China‐Japan Friendship Hospital Beijing China

**Keywords:** Meta‐Analysis, Osteoporotic Vertebral Compression Fractures, Percutaneous Curved Vertebroplasty, Percutaneous Kyphoplasty, Percutaneous Vertebroplasty

## Abstract

This systematic review and meta‐analysis is aimed to provide higher quality evidence regarding the efficacy and safety between PCVP and PVP/KP in OVCFs. We searched the Cochrane Library, PubMed, Web of Science, and Embase databases for all randomized controlled trials (RCTs) and observational studies (cohort or case–control studies) that compare PCVP to PVP/KP for OVCFs. The Cochrane Collaboration's Risk of Bias Tool and Newcastle–Ottawa Scale (NOS) were used to evaluate the quality of the RCTs and non‐RCTs, respectively. Meta‐analysis was performed using RevMan 5.4 software. A total of seven articles consisting of 562 patients with 593 diseased vertebral bodies were included. Statistically significant differences were found in the postoperative visual analog scale (VAS) at 1 day (MD = −0.11; 95% CI: [−0.21 to −0.01], *p* = 0.03), but not at 3 months (MD = −0.21; 95% CI: [−0.41–0.00], *p* = 0.05) or 6 months (MD = 0.03; 95% CI: [−0.13–0.20], *p* = 0.70). There was no statistically significant difference in postoperative Oswestry disability index (ODI) at 1 day (MD = −0.28; 95% CI: [−0.62–0.05], *p* = 0.10), 3 months (MD = −1.52; 95% CI: [−3.11–0.07], *p* = 0.06), or 6 months (MD = 0.18; 95% CI: [−0.13–0.48], *p* = 0.25). Additionally, there were no statistically significant differences in Cobb angle (MD = 0.30; 95% CI: [−1.69–2.30], *p* = 0.77) or anterior vertebral body height (SMD = −0.01; 95% CI: [−0.26–0.23], *p* = 0.92) after surgery. Statistically significant differences were found in surgical time (MD = −8.60; 95% CI: [−13.75 to −3.45], *p* = 0.001), cement infusion volume (MD = −0.82; 95% CI: [−1.50 to −0.14], *P* = 0.02), and dose of fluoroscopy (SMD = −1.22; 95% CI: [−1.84 to −0.60], *p* = 0.0001) between curved and noncurved techniques, especially compared to bilateral PVP. Moreover, cement leakage showed statistically significant difference (OR = 0.40; 95% CI: [0.27–0.60], *p* < 0.0001). Compared with PVP/KP, PCVP is superior for pain relief at short‐term follow‐up. Additionally, PCVP has the advantages of significantly lower surgical time, radiation exposure, bone cement infusion volume, and cement leakage incidence compared to bilateral PVP, while no statistically significant difference is found when compared with unilateral PVP or PKP. In terms of quality of life and radiologic outcomes, the effects of PCVP and PVP/KP are not significantly different. Overall, this meta‐analysis reveals that PCVP was an effective and safe therapy for patients with OVCFs.

## Graphical abstract

This systematic review and meta‐analysis reveal that PCVP is superior for pain relief at short‐term follow‐up compared with PVP/KP. Additionally, PCVP has the advantages of less surgical time, radiation exposure, bone cement infusion volume, and cement leakage incidence than bilateral PVP. Overall, PCVP is an effective and safe therapy for OVCFs.

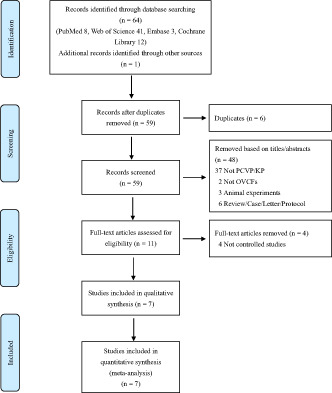

## Introduction

Along with the trend of population aging, osteoporotic vertebral compression fractures (OVCFs) have become a major global public health problem that threatens the health and quality of life of human beings, especially elderly individuals.^1^ It is estimated that the incidence of OVCFs in people aged 50 or older may be 307 cases per 100,000 person years, thus imposing a serious medical and economic burden.^2^, ^3^, ^4^ OVCFs may cause back pain, spinal deformity, limited physiological function, neurological deficits, decreased quality of life, and even increased risk of adjacent vertebral fractures and mortality.^5^, ^6^

Although there are no substantial conclusions on the superiority of cementoplasty over conservative management for OVCFs, it is well‐accepted that conservative treatments are not indicated for every condition and cementoplasty is appropriate in certain circumstances.^7^ Moreover, conservative treatments have inevitable limitations such as residual pain, malunion, prolonged bed rest, and consequent complications. Thus, cementoplasty is strongly recommended for patients with intense pain, severe kyphosis, or substantial functional limitations. Percutaneous vertebroplasty (PVP) involves the percutaneous injection of liquid cement into the collapsed vertebral body by pedicle cannulation. Percutaneous kyphoplasty (PKP) involves the injection of a reduction balloon into the vertebral body, followed by inflation to restore the height of the vertebral body and the injection of bone cement.^8^, ^9^ Vertebral cementoplasty provides rapid pain relief and stabilization with maximum restoration of vertebral height to correct deformities. As such, early mobilization and multisegmental treatment simultaneously are permitted, which is particularly applicable to fragile patients.^10^ Previous studies also confirmed the clinical superiority of PVP/KP compared to conservative treatments,^11^, ^12^ while it must be acknowledged that PVP/KP have limitations. According to an evidenced‐based review,^13^ the symptomatic complication rates of PVP/KP of osteoporotic at 2.2%–3.9%. In the traditional unilateral cementoplasty, cement usually fills only the ipsilateral vertebral bone, and the contralateral vertebral bone is poorly filled. Chen et al.^14^ found that unbalanced cement distribution would lead to an imbalance of stress in the vertebral body, which was related to postoperative biomechanics, pain relief, and refracture.^15^, ^16^ Yu et al.^17^ demonstrated the better clinical outcomes of comparatively diffused pattern. However, increasing the internal inclination angle or the bilateral approach for an even distribution of bone cement may increase the risk of cement leakage, spinal cord injury, and surgical trauma.^18^ Moreover, the bilateral approach requires a long operation time and a large amount of radiation exposure, which is harmful to both the surgeon and the patient. Lonjon^19^ stressed the urgency of radiological safety issues by measuring the irradiation during a cementoplasty procedure: an average of 1.4 ± 2.1 μSv on the entire body with equivalent doses of 44 μSv for the crystalline lens and 59 μSv for the extremities.

Indeed, the best minimally invasive procedure for OVCFs is still controversial, with pros and cons.^20^ Thus, surgeons are being devoted to finding improved procedures, which encourages the creation of novel PCVP. Accordingly, percutaneous curved vertebroplasty (PCVP) was created by changing the head end of the delivery trocar from a flat shape to a variable pivot point at a curved angle, thus taking advantage of Nitinol with ultrahigh elastic properties and polyether ether ketone with the desired mechanical strength. The technique adopted unilateral approach and directional, manipulated, and multiple injection points. Theoretically, the symmetrical and uniform distribution of bone cement can be achieved, which solves the problems caused by a single time, single point injection, excessive amount and uneven distribution of bone cement injection in PVP/KP.^21^, ^22^ Soon et al. indicated that the mean percentage of cement projection across the midline was significantly more in the PCVP group.^22^ Several studies recently investigated the clinical efficacy comparing PCVP versus PVP/KP, however, there is a lack of systematic evidence.^23^ As a novel technology, it is of great importance to reach a clear conclusion for clinical decision‐making. Thus, we conducted this systematic review and meta‐analysis to provide higher quality evidence regarding the efficacy and safety of PCVP compared with PVP/KP in OVCFs.

## Materials and Methods

### Search Strategy

We conducted the current systematic review and meta‐analysis in accordance with the guidelines of the Cochrane handbook for systematic reviews of interventions,^24^ and the latest version of the preferred reporting items for systematic review and meta‐analysis (PRISMA‐P) statement.^25^ The Cochrane Library, PubMed, Web of Science, and Embase databases were searched by two authors (S.Y. and Z.Y.). Our search strategies included “Spinal Fractures” (MeSH Terms), “Osteoporosis” (MeSH Terms) and the keyword for the intervention “Percutaneous Curved Vertebroplasty/Kyphoplasty”, “PCVP/KP”, “curved” or “directional” with the Boolean operators “AND” or “OR”. There was no restriction regarding language and subheadings. We searched the reference lists of the included papers to identify additional studies, and trials not included in the abovementioned databases were screened once they were identified.

### Study Selection

S.Y. and Z.Y. independently screened studies. The inclusion criteria were as follows: (1) published randomized controlled trials (RCTs) and observational studies (cohort or case–control studies); (2) patients with OVCFs; (3) intervention was PCVP/KP compared to PVP/KP.

The exclusion criteria were as follows: (1) OVCFs caused by pathological reasons including tumor, infection, endocrine system disease, etc.; (2) animal experiments; (3) full‐text article could not be obtained.

### Data Extraction

The data processing was independently performed by two authors (S.Y. and Z.Y.) with Endnote X8 software and disagreements were resolved by consulting the third author (M. HN.). The following data were extracted from each study: (1) descriptive statistics such as author information, publication year, study design, country, data sources, t‐score, sample sizes, and vertebral bodies; (2) intervention in experimental and control groups; (3) the outcome of interest: visual analog scale (VAS), Oswestry disability index (ODI), Cobb angle, anterior vertebral body height, surgical time, injected cement volume, duration of fluoroscopy, cement leakage, or refracture.

### Quality Assessment and Risk of Bias

We used the Cochrane Collaboration's Risk of Bias Tool^26^ and the NOS^27^ to evaluate the quality of RCTs and non‐RCTs (cohort or case–control studies) respectively. The Risk of Bias Tool was in the following domains: randomization process, deviations from intended interventions, missing outcome data, measurement of the outcome, and selection of the reported result. We rated each domain as “low”, “some concerns”, or “high”. There were three sections in the NOS: selection, comparability, and outcome. Studies with a score between 0–3 points were considered as low quality, between 4–6 points considered as medium quality, and between 7–9 points considered as high quality.

### Statistical Analysis

The meta‐analysis was performed using RevMan 5.4 software (Cochrane Informatics & Technology Services, United Kingdom). The mean difference (MD) of 95% confidence intervals (CIs) was used for continuous outcomes, while dichotomous variables were reported as odds ratios (ORs) and 95% CIs. Statistical heterogeneity was evaluated utilizing the *I* ^*2*^ test and *P value*. *I* ^*2*^ value less than 25% indicated low heterogeneity and a value less than 50% indicated moderate heterogeneity. In such cases, a fixed effects model was adopted. Otherwise, *I* ^*2*^ value greater than 50% and *P value* less than 0.1 indicated significant heterogeneity, and a random effects model was adopted. If the same outcomes were evaluated by different methods, we applied the standardized mean differences (SMDs) of 95% CIs. If there was a significant heterogeneity, a subgroup analysis was performed by removing studies one at a time. *p* < 0.05 was considered statistically significant.

## Results

### Study Characteristics

**Fig. 1:**
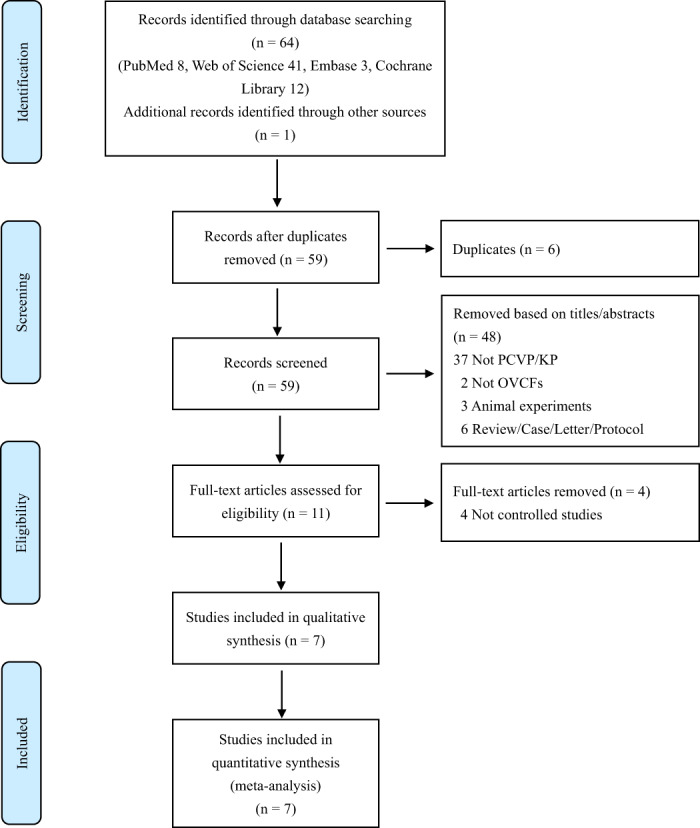
The flow diagram of study selection.

Finally, a total of seven articles including a total of 562 patients with 593 diseased vertebral bodies were included: four RCTs^28^, ^29^, ^30^, ^31^ and three retrospective cohort studies.^32^, ^33^, ^34^ Among them, five studies compared PCVP with bilateral PVP, and three studies compared PCVP with unilateral PVP and one study compared PCVP with bilateral PKP. The study selection process is shown in Figure 1 and the characteristics of the included trials are summarized in Table 1.

**TABLE 1:** Summary of the included studies.

Study	Country	Study design	Sample size		Vertebralbodies (n)		Age (years)		Gender (M/F)		t‐score		Interventions		NOS scores
			I	C	I	C	I	C	I	C	I	C	I	C	
Wang 2021	China	RCT	36	36	36	36	75.55 ± 6.11	76.52 ± 6.24	10/26	12/24	−2.55 ± 0.65	−2.62 ± 0.78	PCVP	BPKP	
Cui 2021	China	Retrospective	23	26	23	26	73.09 ± 6.52	73.25 ± 6.36	6/17	7/19	<−2.5		PCVP	BPVP	*******
Li 2020	China	RCT	35	35	35	35	74.09 ± 8.85	74.89 ± 8.97	6/29	7/28	<−2.5		PCVP	UPVP	
Zhong 2019	China	Retrospective	29	68	35	82	70.7 ± 7.5	73.8 ± 8.2	3/26	12/56	NR		PCVP	BPVP	**********
Cheng 2019^a^	China	RCT	30	26	34	30	71.8 ± 11.2	68.9 ± 11.9	12/18	10/16	−2.9 ± 0.3	−2.8 ± 0.3	PCVP	UPVP	
Cheng 2019^b^	China	RCT	30	22	34	32	71.8 ± 11.2	69.8 ± 12.1	12/18	10/12	−2.9 ± 0.3	−2.9 ± 0.4	PCVP	BPVP	
Huang 2021	China	Retrospective	47	46	47	46	75.5 (61–90)	73.5 (65–87)	12/36	10/36	−3.49 (−4.5–−2.8)	−3.51(−4.0 to −2.7)	PCVP	BPVP	*******
Geng 2021^a^	China	RCT	25	40	25	40	70.7 ± 6.8	70.6 ± 4.8	8/17	14/26	−3.14 ± 0.42	−3.16 ± 0.36	PCVP	UPVP	
Geng 2021^b^	China	RCT	25	31	25	31	70.7 ± 6.8	70.4 ± 6.6	8/17	10/21	−3.14 ± 0.42	−3.23 ± 0.45	PCVP	BPVP	

Note: ^a,b^ Patients were analyzed separately due to multiple control groups.

Abbreviations: BPKP, bilateral percutaneous curved kyphoplasty; BPVP, bilateral percutaneous curved vertebroplasty; C, control group; F, female; I, intervention group; M, male; NR, not reported; PCVP, percutaneous curved vertebroplasty; UPVP, unilateral percutaneous curved vertebroplasty.

### Quality Assessment

**Fig. 2:**
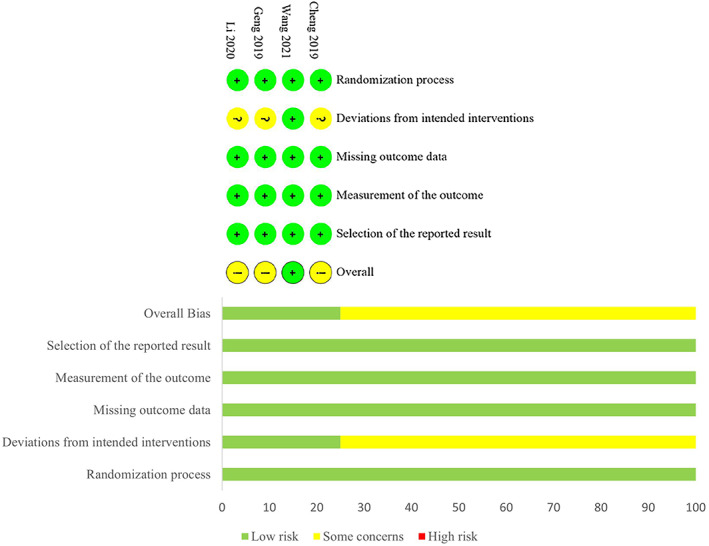
The methodological quality of the included RCTs. Risk of bias summary and risk of bias graph.

As shown in Figure 2, the risk of bias of four RCTs was assessed by the Cochrane Collaboration's Risk of Bias Tool. The randomization process, missing outcome data, measurement of the outcome, and selection of the reported result had low risks of bias in all four RCTs, and deviations from intended interventions had a low risk of bias in one RCT. Three studies were considered to have some concerns regarding deviations from intended intervention due to the characteristics of intervention methods. As shown in Table 1, three cohort studies were assessed according to NOS, and all of them received scores of more than 7.

### Clinical Outcome

#### VAS

**Fig. 3:**
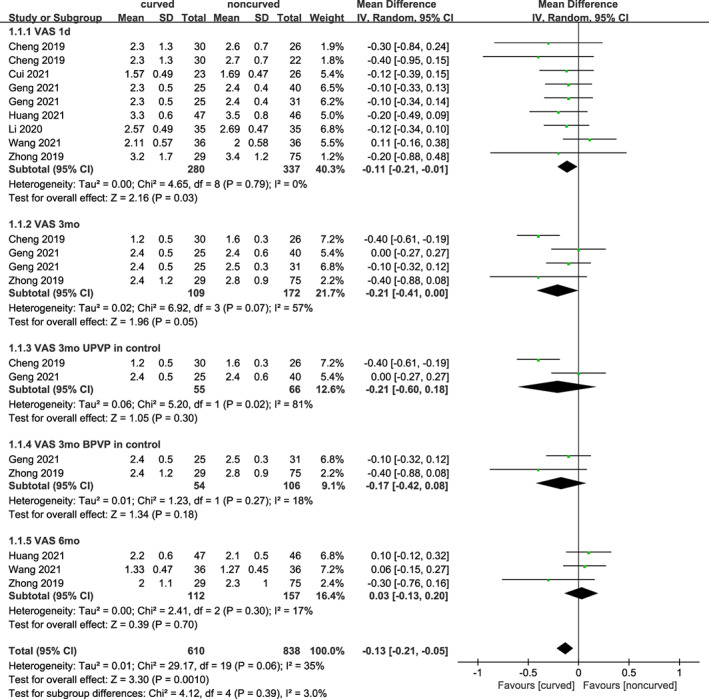
A forest plot depicting the changes in VAS between PCVP/KP and PVP/KP.

Seven trials including 562 subjects reported VAS after surgery at 1 day, three trials including 256 subjects at 3 months, and three trials including 269 subjects at 6 months. As shown in Figure 3, statistically significant differences were found at 1 day [MD = −0.11 (95% CI −0.21 to −0.01), *p* = 0.03, *I* ^*2*^ = 0%] but not at 6 months [MD = 0.03 (95% CI −0.13–0.20), *p* = 0.70, *I* ^*2*^ = 17%] postoperatively. There may be a significant difference 3 months with a high heterogeneity [MD = −0.21 (95% CI −0.41–0.00), *p* = 0.05, *I* ^*2*^ = 57%], thus a further subgroup analyses based on unilateral or bilateral techniques in the control group was performed. However, there was no statistically significant difference regarding unilateral technique [MD = −0.21 (95% CI −0.60–0.18), *p* = 0.30, I^2^ = 81%], or bilateral technique [MD = −0.17 (95% CI −0.42–0.08), *p* = 0.18, I^2^ = 18%] at 3 months. A random effects model was utilized due to low to high heterogeneity.

#### ODI

**Fig. 4:**
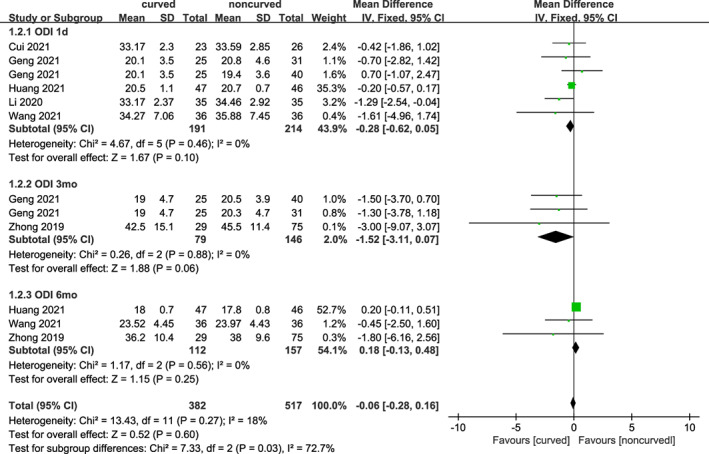
A forest plot depicting the changes in ODI score between PCVP/KP and PVP/KP.

Five trials including 380 subjects reported ODI after surgery at 1 day, two trials including 200 subjects at 3 months, and three trials including 269 subjects at 6 months. As shown in Figure 4, there was no statistically significant difference at 1 day [MD = −0.28 (95% CI −0.62–0.05), *p* = 0.10, *I* ^*2*^ = 0%], 3 months [MD = −1.52 (95% CI −3.11–0.07), *p* = 0.06, *I* ^*2*^ = 0%], and 6 months [MD = 0.18 (95% CI −0.13–0.48), *p* = 0.25, *I* ^*2*^ = 0%] postoperatively. A fixed effects model was utilized due to the lack of heterogeneity.

### Radiographic Measurement

#### Cobb Angle

**Fig. 5:**
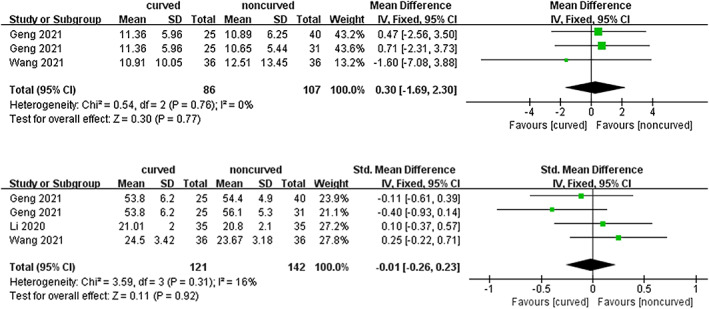
A forest plot depicting radiologic outcomes in the Cobb angle (A) and anterior vertebral body height (B) at final follow‐up between PCVP/KP and PVP/KP.

Two trials including 168 subjects reported Cobb angle at the final follow up after surgery. As shown in Figure 5, there was no statistically significant difference [MD = 0.30 (95% CI −1.69–2.30), *p* = 0.77, *I* ^*2*^ = 0%, Figure 5].

#### Anterior Vertebral Body Height

Three trials including 238 subjects evaluated anterior vertebral body height in different measurements at the final follow‐up after surgery. Additionally, no statistically significant difference was found [SMD = −0.01 (95% CI −0.26–0.23), *p* = 0.92, *I* ^*2*^ = 16%, Figure 5]. A fixed effects model was utilized.

### Surgical Outcome

#### Surgical Time

**Fig. 6:**
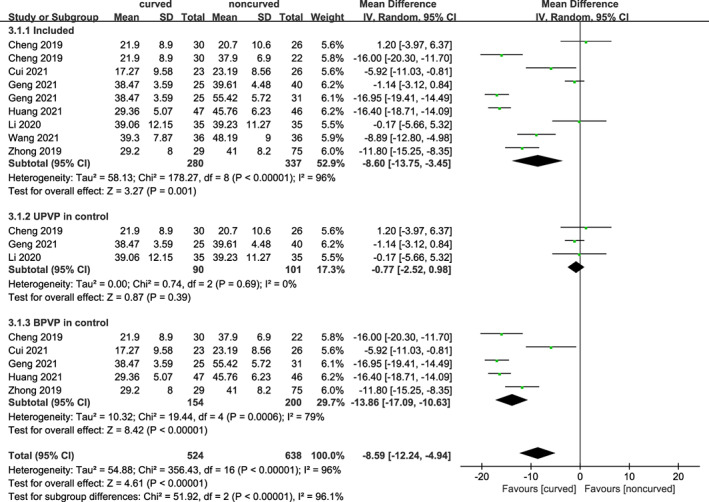
A forest plot depicting surgical time, and subgroup for UPVP and BPVP in control group between PCVP/KP and PVP/KP.

Seven trials including 562 subjects reported surgical time. As shown in Figure 6, a statistically significant difference was found between the curved and noncurved techniques [MD = −8.60 (95% CI −13.75 to −3.45), *p* = 0.001, *I* ^*2*^ = 96%]. Due to high heterogeneity, we performed subgroup analyses based on unilateral or bilateral techniques in the control group. Notably, there were substantial changes in heterogeneity when PCVP was compared to unilateral PVP [MD = −0.77 (95% CI −2.52–0.98), *p* = 0.39, *I* ^*2*^ = 0%] and compared to bilateral PVP [MD = −13.86 (95% CI −17.09 to −10.63), *p* < .00001, *I* ^*2*^ = 79%]. PCVP had the advantage of less surgical time compared to the bilateral PVP, while there was no difference compared to the unilateral PVP.

#### Cement Infusion Volume

**Fig. 7:**
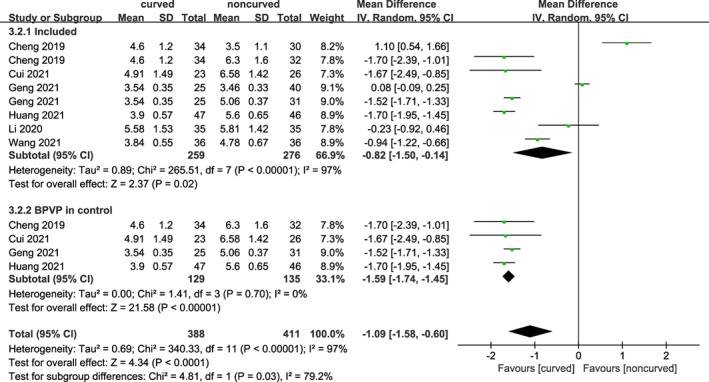
A forest plot depicting infusion volume and subgroup for BPVP in control group between PCVP/KP and PVP/KP.

Six trials including 476 diseased vertebral bodies reported cement infusion volume. As shown in Figure 7, a statistically significant difference was found between the curved and noncurved techniques [MD = −0.82 (95% CI −1.50 to −0.14), *p* = 0.02, *I* ^*2*^ = 97%]. Due to high heterogeneity, we performed subgroup analyses based on unilateral or bilateral techniques in the control group. There were substantial changes in heterogeneity when PCVP compared to bilateral PVP [MD = −1.59 (95% CI −1.74 to −1.45), *p* < 0.00001, *I* ^*2*^ = 0%]. In conclusion, PCVP required less cement infusion volume.

#### Fluoroscopy Doses

**Fig. 8:**
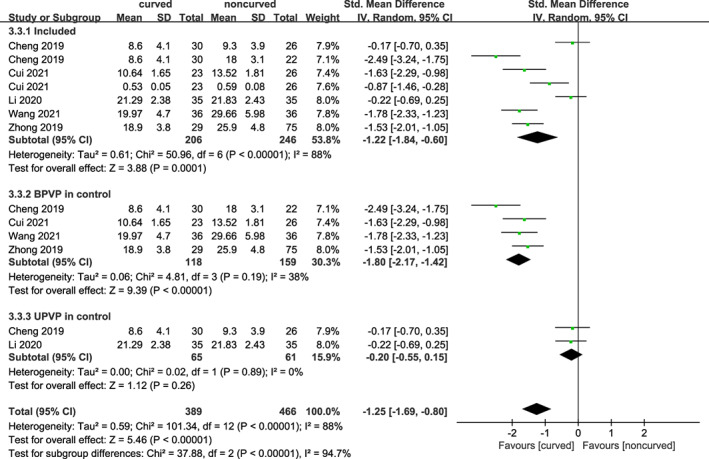
A forest plot depicting duration of fluoroscopy, and subgroup for BPVP and UPVP in control group between PCVP/KP and PVP/KP.

Five trials including 399 subjects reported the dose of fluoroscopy. As shown in Figure 8, a statistically significant difference was found between the curved and noncurved techniques [SMD = −1.22 (95% CI −1.84 to −0.60), *p* = 0.0001, *I* ^*2*^ = 88%]. Additionally, subgroup analyses were performed. There were substantial changes in heterogeneity when PCVP was compared to bilateral PVP [SMD = −1.80 (95% CI −2.17 to −1.42), *p* < 0.0001, *I* ^*2*^ = 38%] and compared to unilateral PVP [SMD = −0.20 (95% CI −0.55–0.15), *p* = 0.26, *I* ^*2*^ = 0%]. Based on the results, PCVP required a lower dose of fluoroscopy than bilateral PVP, while there was no difference compared to unilateral PVP.

### Complications

#### Cement Leakage

**Fig. 9:**
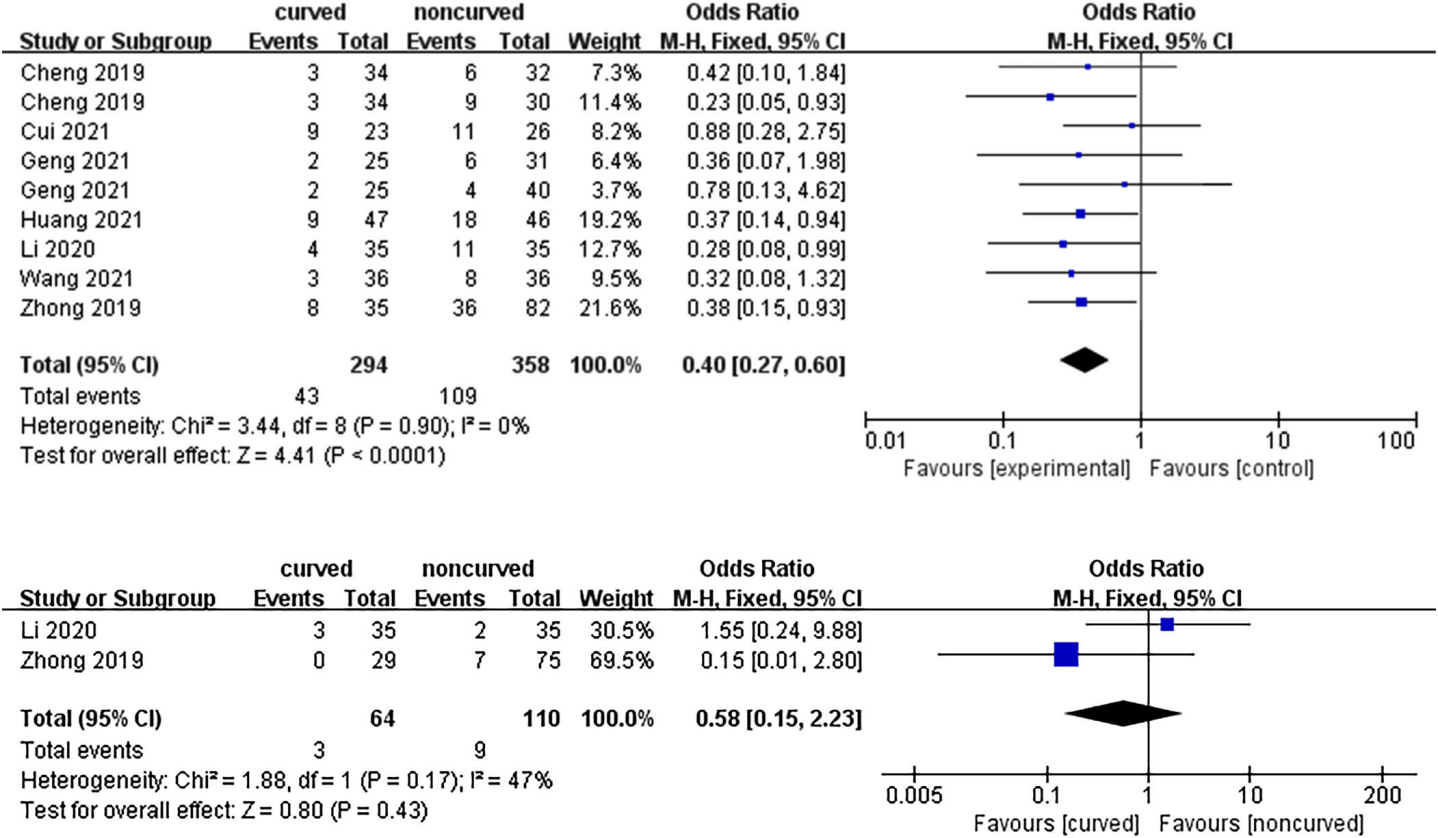
A forest plot depicting postoperative complications regarding cement leakage and refracture between PCVP/KP and PVP/KP.

Seven trials including 562 subjects reported cement leakage. As shown in Figure 9, a statistically significant difference was found between the curved and noncurved technique [OR = 0.40 (95% CI 0.27–0.60), *p* < 0.0001, *I* ^*2*^ = 0%].

#### Refracture

Two trials including 174 subjects reported refracture. There was no statistically significant difference between curved and noncurved techniques [OR = 0.58 (95% CI 0.15–2.23), *p* = 0.43, *I* ^*2*^ = 47%]. A fixed effects model was utilized due to low heterogeneity. Therefore, PCVP had the advantage of a low risk of cement leakage compared to the noncurved technique.

## Discussion

### Clinical and Radiographic Outcome

PCVP showed superior short‐term pain relief compared with conventional cementoplasty. In the present study, statistically significant differences were found in postoperative VAS at 1 day and 3 months, but not at 6 months. As PVP/KP has been widely used, numerous studies have reported the desired analgesic efficacy of PVP/KP maintained for 1 day,^35^ 1 month,^36^ 6 months,^37^, ^38^ 12 months^39^, ^40^ and even 24 months.^41^ As estimated, PVP was effective in relieving pain in 87% of patients.^42^ The underlying mechanism is not fully understood,^43^ partly because set bone cement strengthens the vertebrae and shares trabecular stress, and the thermal effect may damage the nerve endings of the vertebrae and reduce pain.^39^, ^44^ In addition, the uniform distribution of bone cement correlates with the degree of pain relief.^16^ In the process of PCVP, the delivery catheter can be rotated to break the cancellous bone by the fixed front‐end at a curved angle, to obtain better bone cement diffusion.^28^, ^31^ However, mid‐ and long‐term analgesic effects need to be confirmed in the future.

Notably, PCVP showed similar results to PVP/KP regarding ODI, Cobb angle, and anterior vertebral body height at short‐ and mid‐term follow‐up. A multicenter RCT confirmed the long‐term improvement of PKP over conservative treatment with respect to quality of life and kyphotic correction.^45^ However, the correlation between imaging findings and clinical outcomes remains unclear.^46^ Although a recent comparative analysis of kyphoplasty and vertebroplasty found significant differences in anatomical recovery with the former technique slightly superior in long‐term kyphotic correction and vertebral height restoration,^47^ there is no valid quality evidence of balloon kyphoplasty versus vertebroplasty.^20^ Moreover, cementoplasty practically improves quality of life and leads to higher levels of satisfaction, which may benefit from pain relief, a good relationship with the surgeon, and the patient's subjective perceptions.^10^ Thus, a modified evaluation system for multicenter RCTs is warranted to assess the impact on quality of life and radiographic results.

### Surgical Outcome

In view of the similar or better clinical outcomes, PCVP was preferred for safety reasons. According to the results, PCVP had the advantage of less surgical time than bilateral PVP, which is more suitable for elderly patients reluctant to undergo prolonged prone lying.^32^ Due to high heterogeneity, we performed subgroup analyses and there was statistically significant difference when PCVP was compared to bilateral PVP. In addition, PCVP required a lower dose of fluoroscopy than bilateral PVP. Due to massive and potential damage from radiation exposure, protective measures and modified procedures are crucial, especially for cementoplasty, which relies on X‐ray guidance and multiple fluoroscopy during surgery. Orthopaedic surgeons are often exposed to radiation during clinical work, while patients are closer to X‐ray tube and receive a short time and high dose of radiation, which causes more radiation damage during cementoplasty.^48^ A survey of medical staff and patients found that relevant knowledge of radiation exposure was still lacking.^49^ In a retrospective study, the incidence of cancer among orthopaedic surgeons exposed to radiation over a 25‐year period was 29%, compared with 4% among workers without radiation exposure.^50^

### Complications

Regarding complications, PCVP had a lower incidence of cement leakage. Cement leakage could be a serious complication,^20^ correlated with the amount of bone cement injection, the pressure of bone cement perfusion, and rupture of perivertebral cortex.^51^, ^52^, ^53^ In the conventional unilateral approach, the needle needs to be excessively introverted to inject cement into the contralateral side of the vertebral body, which may lead to impairment of the pedicle inner cortical bone and paravertebral nerve injury.^54^ Additionally, unilateral puncture is demanding at the thoracic level, while bilateral approaches may contribute to cement distribution and reduce the rate of epidural cement leakage.^55^ In the present study, PCVP required less cement infusion volume, which is conducive to reducing the consequent risk of cement leakage. In addition, directional and manipulated injection reduced the pressure caused by aggressive cement deposition and the probability of damaging the perivertebral cortex.^32^ Moreover, it was unnecessary to increase the inclination angle of the puncture, which decreased the risk of penetrating the pedicle inner wall and increased the safety of the approach.^29^, ^33^ Theoretically, bilateral injection of bone cement may provide biomechanically better stability through the symmetrical distribution of bone cement in the vertebral body,^16^ while the unilateral approach is prone to asymmetrical distribution of cement. Studies have shown a high correlation between short‐term efficacy, the incidence of long‐term complications, and the degree of bone cement dispersion in the injured vertebral body after PVP.^56^, ^57^ In the present study, Geng et al.^30^ demonstrated a centered cement distribution in PCVPs similar to bipedicular PVP and superior to unipedicular PVP. However, there was no difference in refracture between PCVP and cementoplasty. Previous studies confirmed that advanced age, lower bone mineral density, female sex, and low estradiol concentrations were mainly associated with the risk of refracture after PVP.^57^, ^58^

### Limitations

To the best of our knowledge, this was the first meta‐analysis to evaluate the effectiveness of PCVP versus PVP/KP on OVCFs. However, the study had some limitations. First, the PCVP methods varied among studies, which may increase the risk of heterogeneity. Second, most studies included OVCFs at a single level due to the complex situation in multiple OVCFs. The combination of several techniques may be an alternative under certain circumstances. Third, the number of studies included was limited, partly due to the initial application stage of the novel technique. Therefore, more RCTs with comprehensive and long‐term clinical and imaging results are needed in the future to better determine efficacy and facilitate standardized treatment regimens.

### Conclusions

Compared with PVP/KP, PCVP is superior for pain relief at short‐term follow‐up. Additionally, it has the advantages of significantly lower surgical time, radiation exposure, and bone cement infusion volume and cement leakage incidence than bilateral PVP, while no statistically significant difference is found when compared with unilateral PVP or PKP. In terms of quality of life and radiologic outcomes, the effects of PCVP and PVP/KP are not significantly different. Overall, this meta‐analysis reveals that PCVP was an effective and safe therapy for patients with OVCFs.

## Conflict of Interest Statement

The authors declare that they have no conflict of interest.

## Ethical Approval

This study is a meta‐analysis and does not require ethical approval and consent to participate.

## Authors' Contributions

S.Y. and Z.Z.H. conceived and designed the study. S.Y. and Z.Y. performed the literature search and collected the data. T.M.S. and M.H.N. contributed to the critical revision of the manuscript. All authors were involved in writing the manuscript. All authors read and approved the final manuscript.

## Data Availability

The datasets used and/or analyzed during the present study are available from the corresponding author upon reasonable request.
